# Multiple evolutionary origins of glyphosate resistance in *Lolium multiflorum*

**DOI:** 10.1111/eva.13344

**Published:** 2022-02-08

**Authors:** Caio A. C. G. Brunharo, Matthew A. Streisfeld

**Affiliations:** ^1^ 2694 Department of Crop and Soil Science Oregon State University Corvallis Oregon USA; ^2^ 3265 Institute of Ecology and Evolution University of Oregon Eugene Oregon USA

**Keywords:** ABC transporter, admixture, EPSPS, glyphosate resistance, herbicide resistance, *Lolium*, repeated evolution, weed evolution

## Abstract

The multitude of herbicide resistance patterns that have evolved in different weed species is a remarkable example of the rapid adaptation to anthropogenic‐driven disturbance. Recently, resistance to glyphosate was identified in multiple populations of *Lolium multiflorum* in Oregon. We used phenotypic approaches, as well as population genomic and gene expression analyses, to determine whether known mechanisms were responsible for glyphosate resistance and whether resistance phenotypes evolved independently in different populations, and to identify potential loci contributing to resistance. We found no evidence of genetic alterations or expression changes at known target and non‐target‐site resistance mechanisms of glyphosate. Population genomic analyses indicated that resistant populations tended to have largely distinct ancestry from one another, suggesting that glyphosate resistance did not spread among populations by gene flow. Rather, resistance appears to have evolved independently on different genetic backgrounds. We also detected potential loci associated with the resistance phenotype, some of which encode proteins with potential effects on herbicide metabolism. Our results suggest that Oregon populations of *L*. *multiflorum* evolved resistance to glyphosate due to a novel mechanism. Future studies that characterize the gene or genes involved in resistance will be necessary to confirm this conclusion.

## 1 INTRODUCTION

The human population is expected to reach nearly 10 billion by 2050, and meeting agricultural demands remains one of the biggest challenges for our society (United Nations, 2019). Weed interference can significantly reduce crop yields (Appleby et al., 1976; Oerke, 2006). Weeds of agricultural crops are primarily managed with herbicides, as other techniques are less efficient and typically more expensive. The over‐reliance on herbicides as the main weed management tool in agriculture has selected for herbicide‐resistant weed populations. To date, 509 examples of herbicide resistance have been reported (Heap, 2021) from over 90 different crops and 70 countries around the world. Herbicide resistance poses a serious challenge for sustainable weed management, because new herbicides have not been developed for marketing in recent years (Duke, 2012), and there are numerous additional costs associated with nonchemical control methods (Swinton & Deynze, 2017). Furthermore, in some situations, fields with a long history of no‐tilling that contain herbicide‐resistant weeds may have to return to conventional weed control techniques, increasing the carbon footprint of food production and moving against basic concepts of sustainable agriculture (Pretty, 2018).

Herbicides pose a strong selection pressure on weed populations, as control levels typically reach >95% (Diggle et al., 2003). Given the widespread use of herbicides in conventional agriculture, herbicide resistance is now an issue in many agroregions throughout the world. The rapid and repeated evolution of herbicide resistance in agricultural fields is a clear example of parallel evolution due to the presence of similar selective pressures (Bolnick et al., 2018). There are many examples where the same genetic change resulted in the evolution of herbicide resistance across distinct plant lineages. To illustrate, over 90 populations from 40 different weed species have a mutation at position 197 of acetolactate synthase (ALS), conferring resistance to different herbicides that inhibit this enzyme (Heap, 2021). Despite this clear convergence on a single mutation site, in some cases the genetic basis of herbicide resistance may involve multiple mechanisms: Mutations can occur in the target site of the herbicide (denominated target‐site resistance, TSR) or elsewhere (non‐target‐site resistance, NTSR) (Baucom, 2019). TSR has been demonstrated in several plant systems, and the functional basis of these mechanisms has been widely elucidated. A well‐studied example comes from mutations in the gene that encodes enolpyruvylshikimate‐3‐phosphate synthase (EPSPS). This is a key enzyme in the shikimate pathway that is crucial for aromatic amino acid biosynthesis. The activity of EPSPS is inhibited by the herbicide glyphosate at the phosphoenolpyruvate binding site (Steinrücken & Amrhein, 1984). Amino acid substitutions in the active site of the enzyme, specifically at position 106, have been shown to provide reduced glyphosate binding due to structural conformation changes (Funke et al., 2006). This mutation does not dramatically reduce the catalytic efficiency of EPSPS in the presence of glyphosate, allowing treated plants to survive. TSR may also be conferred by deletions in the herbicide target site, as is commonly found for protoporphyrinogen oxidase inhibitors (Patzoldt et al., 2006), or because of duplication of the target‐site gene (Gaines et al., 2010). In the latter scenario, increased target‐site expression is conferred by a higher concentration of the enzyme in the plant cells, which in turn requires a substantially higher concentration of herbicide to be absorbed, which is often impractical.

NTSR mechanisms are classified as those not involving the herbicide target site. The physiological bases of these mechanisms have been broadly described. In general, weeds with NTSR may exhibit reduced herbicide uptake, reduced translocation to the site of action, and enhanced herbicide degradation (reviewed by Delye et al., 2013). In recent years, many researchers have focused on elucidating the pathways involved in herbicide degradation (i.e., metabolic resistance), and results suggest the involvement of cytochrome P450s and glutathione S‐transferases. Most conclusions, however, come from indirect evidence, such as the application of P450 inhibitors followed by herbicide treatment (Oliveira et al., 2018), which would reverse resistance if enhanced activity of P450 is responsible for the resistance phenotype. However, the genes involved in NTSR are still largely unknown (Han et al., 2021). The primary reason for the recent increased interest in metabolic herbicide resistance is the observation that weed populations with these types of resistance mechanisms commonly exhibit resistance to multiple herbicides from different chemical groups and mechanisms of action (Dimaano et al., 2020). Therefore, NTSR may confer resistance to weed populations against herbicides they have never been exposed to (Busi & Powles, 2016).

Although TSR is believed to be conferred by single major‐effect alleles and likely contributes to much of the parallelism in resistance phenotypes among weed species, NTSR is believed to be a quantitative trait, conferred by multiple loci of small effect (Delye, 2013; Delye et al., 2013). However, recent research has shown that the NTSR phenotype can be explained by major‐effect genes, such as enhanced expression of the P450 *CYP81A10v7* that conferred resistance to seven herbicide chemistries (Han et al., 2021). While physiological modifications that lead to NTSR in weeds are well documented, their genetic basis remains poorly understood (Suzukawa et al., 2021).

*Lolium multiflorum* L. is a diploid (*2n* = *2x* = 14), obligate outcrossing winter annual species of broad occurrence in the United States (USDA‐NRCS, 2021) and throughout the world. It is native to the Mediterranean basin, and because of its desirable forage characteristics, this species has been adopted as a crop in many regions of the world (Humphreys et al., 2010). Although *L*. *multiflorum* is cultivated as a crop, this species is also considered a weed when it grows where it is not desired. For instance, commercial *L*. *multiflorum* varieties are often sown as cover crops in the United States to enhance soil health indices in corn–soybean rotations (Shipley et al., 1992). However, persistence of *L*. *multiflorum* in subsequent growing seasons is not desirable, because it could compete with the cash crop early in the season. For clarity, hereafter, we refer to “annual ryegrass” when discussing the crop, and *L*. *multiflorum* when describing the weed. The cultivated varieties of annual ryegrass, although the same species as the weedy biotypes, exhibit desirable traits associated with yield, including high nitrogen content and high seed vigor, and they are susceptible to herbicides.

*L* *olium* *multiflorum* has evolved resistance to many herbicides that operate by a variety of molecular mechanisms (Suzukawa et al., 2021). For example, a population collected from a prune orchard in California exhibited resistance to four herbicides that act by different mechanisms of action (Brunharo & Hanson, 2018). In Oregon, *L*. *multiflorum* is a weed in many crops, including perennial ryegrass, tall fescue, wheat, orchardgrass, and annual ryegrass grown for seed. Recently, 60 populations of *L*. *multiflorum* in Oregon were identified to be resistant to herbicides, with some populations resistant to as many as four different mechanisms of action (Bobadilla et al., 2021). The widespread herbicide resistance in grass seed fields in Oregon poses a serious threat to local agricultural communities, because of potential contamination of grass seed lots with herbicide‐resistant weed seeds. The seed lots are sold for many uses nationally and internationally, posing a potential source for dispersal of herbicide resistance genes.

Basic knowledge of weed adaptation to herbicides, dispersal, and detection is of primary importance to initiate a mitigation plan and to prevent expansion of the areas infested with herbicide resistance. Gene flow from herbicide‐resistant weed populations into annual ryegrass crops is one of the main concerns of farmers, and its spread is commonly attributed to movement of agricultural machinery, commodity movement, and livestock feed (Schroeder et al., 2018). Understanding the genetic relatedness among herbicide‐resistant and herbicide‐susceptible weed populations can offer clues to potential mechanisms of propagule dispersal. For example, if a resistant weed population spreads through seed lot contamination, then policymakers could implement new rules or recommend new practices to prevent seed lot contamination (e.g., recommend seed certification based on herbicide resistance testing of seed lots, recommend longer rotations in the field, and others). In addition, understanding the origins of herbicide resistance evolution could better assist farmers manage herbicide resistance before it spreads. For instance, if herbicide resistance is more likely to be selected locally, then farmers should manage weeds locally to prevent seed set.

In herbicide‐resistant populations of *L*. *multiflorum* in Oregon, little is known about the underlying genetic mechanisms conferring herbicide resistance, or the genetic relationships among populations. In this study, we analyze patterns of genetic variation and admixture among 16 of these Oregon populations that vary in their resistance to glyphosate. The three primary objectives of this work were to (1) determine whether resistance is conferred by known resistance mechanisms, (2) determine whether resistance phenotypes evolved independently in different populations, and (3) identify potential loci involved in glyphosate resistance.

## 2 MATERIALS AND METHODS

### 2.1 Study populations

**FIGURE 1:**
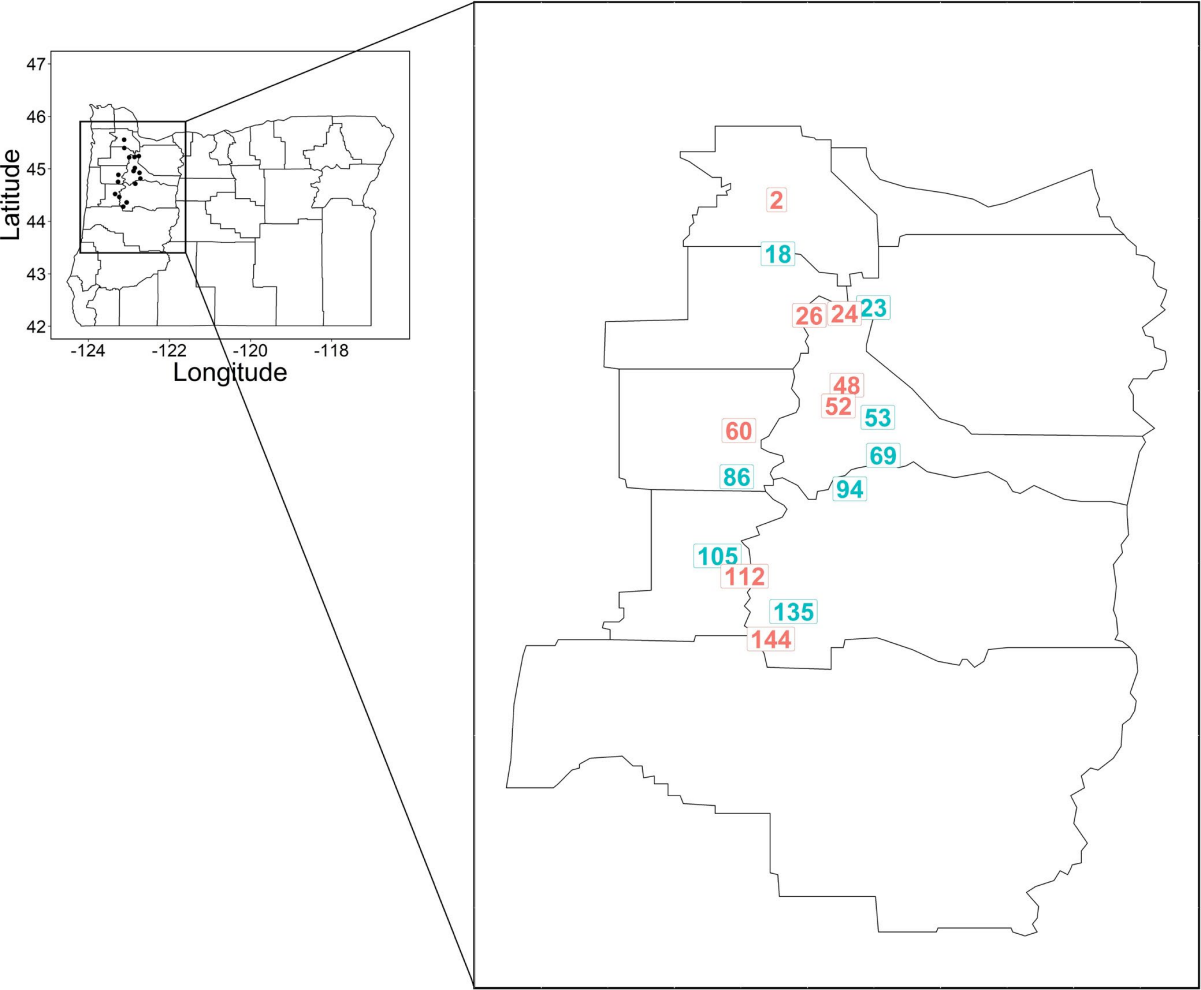
Collection sites of the 16 Oregon *Lolium multiflorum* populations used in this study

A set of 16 *L*. *multiflorum* populations from agricultural fields in the Willamette Valley in Oregon were identified for this study (Figure 1, Table S1). The populations were collected in 2017–2018 as part of a broader survey of herbicide resistance (Bobadilla et al., 2021). From each field, seeds from 25–30 mature plants were collected and later pooled in approximately equal amounts. A cultivated, public variety of annual ryegrass known as “Gulf” and a previously characterized multiple herbicide‐resistant *L*. *multiflorum* population called PRHC from California (Brunharo & Hanson, 2018) were included in the study, as was a cultivated variety of perennial ryegrass (*L*. *perenne* L.) used as an outgroup. Gulf has been used widely as a reference susceptible population for *L*. *multiflorum* herbicide resistance characterization (Bobadilla et al., 2021), whereas PRHC is a population that exhibits resistance to four different herbicide mechanisms of action, including a known target‐site mutation in *EPSPS*. The populations selected exhibited various herbicide resistance patterns, where resistance to ALS (mesosulfuron and pyroxsulam), acetyl‐CoA‐carboxylase (ACCase; clethodim, pinoxaden, and quizalofop), and EPSPS (glyphosate) inhibitors was the most common. Populations susceptible to all herbicides tested were also included in the study. However, in this study, we focused only on glyphosate because of its importance in agricultural and noncropping areas, and because the NTSR mechanisms of resistance are largely unknown.

### 2.2 Phenotyping

To confirm glyphosate resistance in the sampled populations, we performed a shikimate accumulation assay (see Table S1 for number of samples evaluated from each site). This method was implemented as a biomarker for glyphosate effects, because glyphosate inhibits EPSPS in the shikimate pathway, resulting in the accumulation of shikimate in the tissues (Dayan et al., 2015). Because this assay is quantitative, we were able to accurately diagnose the level of glyphosate resistance in each sampled individual across populations. If shikimate accumulation levels are distributed continuously within and among populations, then it would suggest quantitative control of glyphosate resistance, likely due to the contribution of multiple genetic loci. Conversely, qualitative levels of shikimate accumulation likely indicate a more simple genetic basis for glyphosate resistance. Field‐collected seeds were germinated in petri dishes, and seedlings were transplanted to 0.5‐L pots filled with commercial potting mix and grown in a greenhouse at 24°C and 14/10 h (day/night). Twenty plants were grown from each *L*. *multiflorum* population. When plants reached the 23‐BBCH (three tillers) growth stage (Hess et al., 1997), leaf tissue from the second youngest fully expanded leaves was collected, frozen in liquid nitrogen, and stored in a −80°C freezer until further analysis. After tissue sampling, glyphosate was applied at 1456 g acid equivalent per hectare (g a.e. ha^−1^), which is within the recommended field rate for most crops where glyphosate is used.

Forty‐eight hours after glyphosate application, the youngest fully expanded leaves from a different tiller were collected, weighed, and stored in Eppendorf tubes in a −80°C freezer until shikimate accumulation quantification was performed as described in Shaner et al. (2005). Briefly, samples were pulverized in liquid nitrogen, and 1000 µl of 10 mM ammonium phosphate monobasic (0.1% Tween, pH 4.4 with 0.1 HCl or NaOH) was added to the ground tissue. To enhance cell lysis, two freeze–thaw steps were performed by freezing samples in a −20°C freezer for 2 h, and thawing at 60°C for 1 h. Then, 250 µl of 1.25 N HCl was added to each sample and incubated at 60°C for 15 min. A 25 µl aliquot was transferred to microtiter plates, and 100 µl of 0.25% (w/v) periodic acid and 0.25% (w/v) sodium *m*‐periodate were added. Samples were incubated at room temperature for 90 min. To stop shikimate oxidation, 100 µl of 0.6 N NaOH and 0.22 M sodium sulfite were added. Shikimate was quantified at 380 nm using a spectrophotometer, and data were analyzed by fitting a standard curve of technical grade shikimate and subtracting background absorbance from samples. Data are presented in ng shikimate µg^−1^ fresh weight (FW). Survival data were also collected 30 days after glyphosate treatment by giving a “0” for plants that survived, and “1” to individuals that died.

### 2.3 High‐throughput sequencing

A genotype‐by‐sequencing study was performed to identify SNPs for population genetic analyses. From each of the 16 Oregon populations, as well as the Gulf, PRHC, and *L*. *perenne* sites, we included a mean of 15 samples per site (range 13–16 samples). DNA was extracted with a commercial kit following the manufacturer's recommendations (Mag‐Bind^®^ Plant DNA DS, Omega Bio‐Tek), followed by sample preparation according to the method developed by Elshire et al. (2011). Briefly, 200 ng of DNA from each sample was digested with 10 U *ApeKI* (New England BioLabs Inc.) for 2 h at 75 C. Barcodes (4–8 bp) and common adapters were ligated with 400 U *T4 DNA ligase* (New England BioLabs Inc.) at 22°C for 1 h, followed by enzyme inactivation at 65 C for 30 min. Samples were then multiplexed (96 samples per pool, total of three pools) and purified using a commercial kit (QiAquick PCR Purification Kit, Qiagen). A PCR amplification step was performed using P1 and P2 as primers with 14 cycles (98C for 30 s, 14 cycles of 98°C for 10 s, 68°C for 30 s, and 72°C for 30 s) and a final extension at 72°C for 5 min (Phusion High‐Fidelity PCR Master Mix, Thermo Scientific). A final library clean‐up step was performed before sequencing (QiAquick PCR Purification Kit, Qiagen). Library quality control was performed with qPCR and a bioanalyzer (High Sensitivity DNA Analysis, Agilent). Sequencing (three libraries of 96 multiplexed samples each) was performed with a HiSeq 3000 in 150‐bp paired‐end mode at the Center for Genome Research and Biocomputing (CGRB) at Oregon State University.

High‐throughput sequencing data were processed with Stacks 2.55 (Rochette et al., 2019). Samples were demultiplexed with the *process_radtags* module, with the ‐‐*paired*, ‐*c*, ‐*q*, and ‐*r* flags. *De novo* assembly of loci was optimized as recommended by Paris et al. (2017). First, forward reads were assembled *de novo* with the *ustacks* module (*M* = 4, *m* = 3, *N* = 2). Second, *cstacks* was used to build catalog loci. Third, *sstacks* was used to align the *de novo* loci to the catalog. Fourth, *tsv2bam* was implemented to transpose sequencing data to be oriented by locus, and paired‐end reads were integrated to each single‐end locus assembled. Finally, SNPs were called with the *gstacks* module. An integrated approach was used to align the catalog loci to the draft genome of *Lolium perenne*, a close relative to *L*. *multiflorum* (Byrne et al., 2015). We integrated the alignments back into the *gstacks* files using the *stacks*‐*integrate*‐*alignments* program and obtained genomic coordinates. Building *de novo* loci, followed by alignment of consensus sequences to the draft genome of *L*. *perenne*, combines the advantages of the *de novo* approach with the positional data from the draft genome (Paris et al., 2017).

SNPs were filtered with the *populations* module of Stacks (‐‐*min*‐*maf* 0.05, ‐‐*max*‐*obs*‐*het* 0.7), followed by *vcftools* (Danecek et al., 2011) to retain biallelic sites (‐‐*min*‐*alleles 2* ‐‐*max*‐*alleles 2*), and exclude sites that were missing in more than 50% of the individuals (‐‐*max*‐*missing 0*.*5*) This dataset was used for the population genetic analyses described below.

### 2.4 Sanger sequencing

Amino acid substitutions in EPSPS at position 102 and/or 106 have been demonstrated to cause conformational changes in the glyphosate target enzyme, preventing inhibition (reviewed by Sammons & Gaines, 2014). We used Sanger sequencing to test whether these previously identified mutations in the *EPSPS* gene also played a role in glyphosate resistance in the Oregon populations of *L*. *multiflorum*. DNA from 5–6 samples per population was used to amplify a 338‐bp fragment of the *EPSPS* gene containing positions 102 and 106, which is located in exon 2 of this 1536‐bp‐long gene (based on the coding sequence of *Oryza sativa*). We used primers described by Adu‐Yeboah et al. (2014) for PCR amplification (Platinum *Taq* DNA Polymerase High Fidelity, Invitrogen) following the manufacturer's recommendations. BigDye Terminator v3.1 (Applied Biosystems, Beverly, MA, USA) was used for sequencing in an ABI 3730 sequencer (Applied Biosystems Inc.). To determine whether resistant individuals were differentiated from susceptible individuals, we performed a multiple alignment in Geneious Prime 2020.0.4 (www.geneious.com) with a reference *EPSPS* sequence from *L*. *multiflorum* (Perez‐Jones et al., 2007).

### 2.5 Copy‐number variation and gene expression analysis

Copy‐number variation of *EPSPS* has been identified to confer glyphosate resistance in several weed populations, including *L*. *multiflorum* from Arkansas (Salas et al., 2012). More recently, an ABC transporter was shown to be constitutively up‐regulated in the weed *Echinochloa colona* (L.) Link (Pan et al., 2021). This transporter is localized to the plasma membranes and is believed to be involved in the efflux of glyphosate from the cytoplasm into the apoplast.

Primers were designed to amplify a 68‐bp fragment in the coding region of *EPSPS* from the *L*. *multiflorum* populations from our study (Table S2), as well as a 135‐bp fragment of *ALS* that was chosen as a housekeeping gene (Brunharo et al., 2019; Dillon et al., 2017). Five samples were analyzed from each of the resistant populations and two susceptible populations (“Gulf” and “lm_105”). We used genomic DNA for the *EPSPS* copy‐number variation analysis. Reactions consisted of 5 µl of SsoAdvanced Universal SYBR^®^ Green Supermix (Bio‐Rad), 0.25 µl of forward and 0.25 µl of reverse primer at 10 µM, and 2 µl of genomic DNA normalized to 5 ng µl^−1^, and were performed in a StepOnePlus™ qRT‐PCR System (Applied Biosystems Inc.). Amplification was carried out with an initial denaturation cycle at 98°C for 3 min, and 40 cycles of 98°C for 15 s, followed by 64°C for 60 s. Melt curves were generated to assess specificity of primers, and reaction products were run in a 1% agarose gel to assess fragment size and number. Primer efficiency was also performed with both primer sets. *EPSPS* copy number from resistant populations, as well as lm_105, was compared with Gulf.

For quantifying the gene expression of the ABC transporter gene, *ABCC8*, we designed several primer pairs to amplify a region of this gene based on the available sequence from *E*. *colona* (NCBI accession number MT249005.1). After Sanger sequencing a 420‐bp fragment from *L*. *multiflorum*, we designed shorter, *L*. *multiflorum*‐specific primers for quantitative real‐time PCR (Table S2). *ALS* was also used as a housekeeping gene to normalize the expression levels of *ABCC8*. Approximately 50 mg of leaf tissue was sampled from plants at the 3‐leaf stage and immediately frozen in liquid nitrogen. Five samples were analyzed from each of the resistant populations and two susceptible populations (“Gulf” and “lm_105”). RNA was extracted using a commercial kit (RNeasy Plant Mini Kit, Qiagen), followed by cDNA synthesis (iSCRIPT, Bio‐Rad). The qRT‐PCR was performed as described for *EPSPS*, with three technical replicates. The experimental runs were pooled into one dataset based on a Levene test of homogeneity of variance (*p* = 0.64). Copy‐number variation and gene expression were quantified using the $2^{‐\Delta \Delta C_{t}}$ method (Schmittgen & Livak, 2008), and multiple comparisons were performed using Tukey's contrasts (*glht* function) in R with a Bonferroni correction considering 10 populations. *ABBC8* gene expression from resistant populations, as well as lm_105, was compared with Gulf.

### 2.6 Patterns of population genetic variation

To quantify levels of genetic variation within populations, we used *stacks* to compute the nucleotide diversity (π), expected heterozygosity (H*_E_*), observed heterozygosity (H*_O_*), and F_IS_ (inbreeding coefficient) for each population. To test whether levels of genetic diversity were different between resistant and susceptible populations, we used a two‐sample Wilcoxon test in R for each measure of genetic variation.

Principal component analysis (PCA) was implemented to obtain an overview of the population structure among samples. PCA is a model‐free data summary technique, enabling the identification of population structure regardless of the historical underlying process shaping present levels of genetic variability (McVean, 2009). Separate analyses were performed that included (a) the entire dataset, containing Oregon populations, Gulf, the California population, and perennial, and (b) the Oregon populations only. The *prcomp* function was applied to a scaled SNP dataset using a custom R script, and eigenvalues were plotted with ggplot2 (Wickham, 2016).

To further dissect the historical demographic events in the study populations, we inferred patterns of ancestry and admixture using ADMIXTURE 1.3.0 (Alexander et al., 2009). We again ran the analyses using two different datasets, similar to the PCA: The first consisted of all sampled populations (including Gulf, PRHC, perennial, and Oregon populations), and the second, only the Oregon populations. Prior to analysis, the *vcf* was sorted and converted to HapMap format using TASSEL (Bradbury et al., 2007), before further conversion to a binary format with PLINK (Purcell et al., 2007). ADMIXTURE was run with multiple values of *K* (1–10) as outlined in Liu et al. (2020), and the *Q* scores reflecting the probability of assignment of each individual to cluster *K* from the two analyses were plotted with PONG (Behr et al., 2016). Under a scenario where resistance evolved once from a single common ancestor and then spread throughout the region, all resistant samples should show similar patterns of ancestry. However, if resistance evolved on multiple genetic backgrounds independently, then resistant samples should show little grouping at different levels of *K*.

In an attempt to identify genomic regions under selection, we performed an F_ST_ analysis between each pair of resistant and susceptible populations. Given the clear adaptive benefit of glyphosate resistance, natural selection at resistance loci should result in locally elevated genetic divergence between resistant and susceptive populations. Moreover, if resistance was conferred by a single locus that was shared among all resistant populations, then the same highly differentiated locus should be observed in comparisons between all resistant and susceptible populations. Conversely, if multiple F_ST_ outliers are observed, then this suggests a more complex genetic architecture is involved, with potentially distinct resistance mechanisms and independent origins.

Genome‐wide pairwise F_ST_ values were obtained with the ‐‐*fstats* flag of the *populations* module in Stacks. For this analysis, we applied a more relaxed filtering step to maximize the number of SNPs distributed throughout the genome. In the *populations* module of Stacks, we included the ‐‐*min*‐*maf* 0.05, and the ‐‐*max*‐*obs*‐*het* 0.7 flags followed by ‐‐*max*‐*missing 0*.*1*, ‐‐*min*‐*alleles 2*, and ‐‐*max*‐*alleles* 2 in vcftools. We then applied LinkImputeR (Money et al., 2017) to impute missing genotypes. The SNP dataset was subjected to 20 combinations of filters and read depths to assess accuracy and aid in the decision of imputation parameters. A similar approach to impute missing genotypes prior to identifying loci involved in local adaptation was adopted elsewhere (Colque‐Little et al., 2021). For all possible comparisons between each resistant and susceptible population, we extracted the top 1% of the F_ST_ distribution and searched for loci that were found to overlap among multiple comparisons.

### 2.7 Outlier annotation

To determine potential loci involved in glyphosate resistance, we annotated the genomic contigs containing outlier loci. For all loci that were found in common in the top 1% of the most differentiated SNPs between a single resistant population and each of the susceptible populations, we extracted the entire contig containing that site from the *L*. *perenne* draft genome (Byrne et al., 2015). Augustus was used to predict genes within these contigs using an *Arabidopsis thaliana* trained dataset (Stanke et al., 2006). Predicted genes were annotated with Blast2GO 5 (Götz et al., 2008) using the *nr* database from NCBI, with an E‐value cutoff of 10^−10^.

## 3 RESULTS

### 3.1 Glyphosate resistance is widespread

**FIGURE 2:**
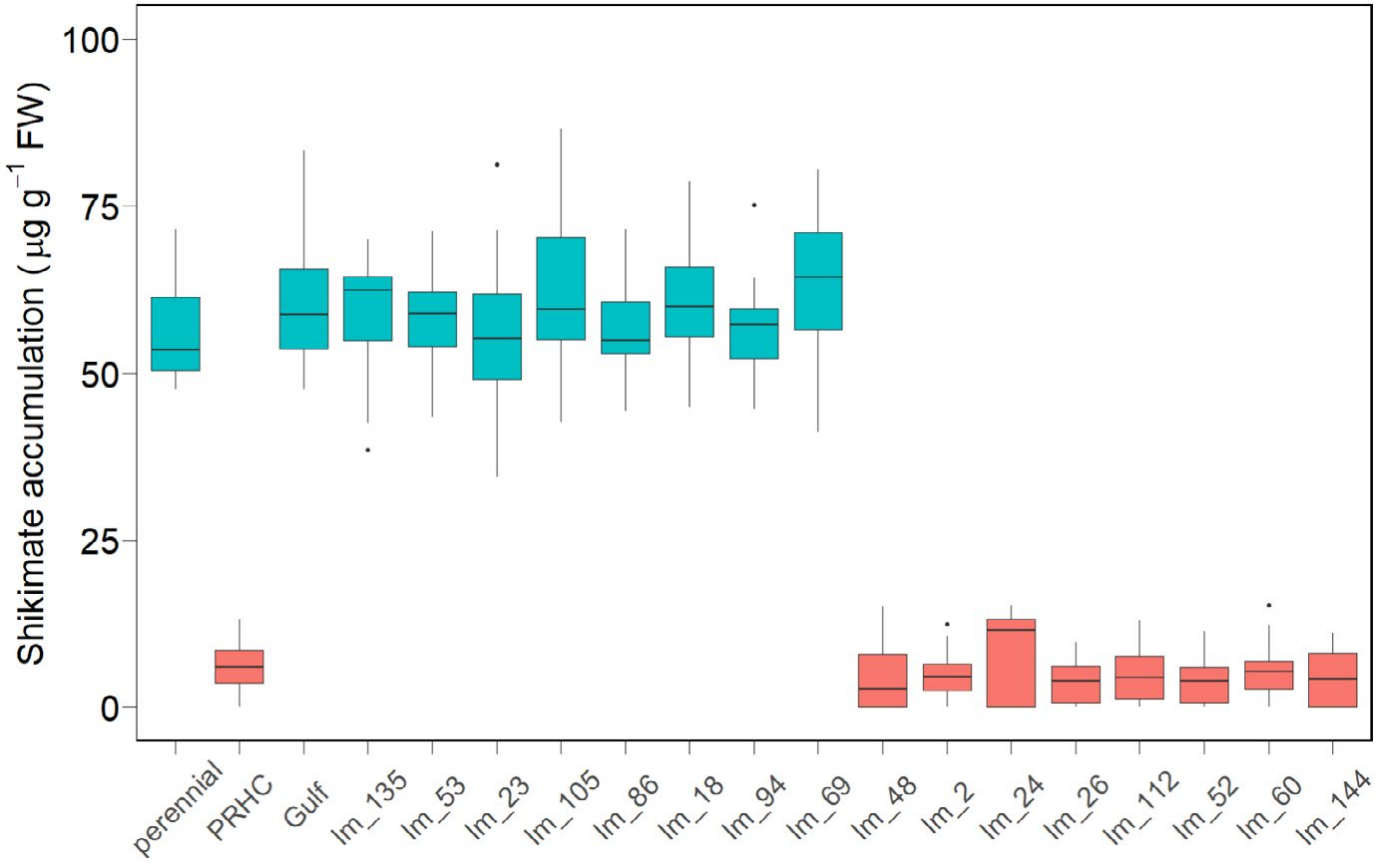
Shikimate accumulation in glyphosate‐resistant and glyphosate‐susceptible *L*. *multiflorum* 48 hours after glyphosate treatment at 1456 g e.a. ha^−1^. Horizontal lines correspond to the median accumulation, box heights indicate the lower and upper quartile, and whiskers correspond to 1.5 times the interquartile range. High shikimate accumulation indicates high susceptibility to glyphosate treatment

Our approach to phenotype *L*. *multiflorum* plants provided a clear distinction between resistant and susceptible samples, because the 1456 g e.a. ha^−1^ glyphosate dose killed all individuals from the known susceptible population (Gulf), whereas 100% survival was observed in the known glyphosate‐resistant population (PRHC). Out of the 16 Oregon field populations analyzed, eight were glyphosate‐resistant and were characterized by an exceptionally low accumulation of shikimate (Figure 2). Susceptibility or resistance to glyphosate based on shikimate accumulation was largely uniform among samples within populations (Table S1). Susceptible samples consistently accumulated >50 µg g^−1^ FW of shikimate, whereas resistant plants accumulated <10 µg g^−1^ FW. The qualitative nature of shikimate accumulation between resistant and susceptible populations suggests there is a simple genetic basis for glyphosate resistance. The shikimate accumulation data were highly consistent with data on survival, with mortality almost always occurring in individuals that accumulated high levels of shikimate. For a single sample in populations lm_24 and lm_60, and two each from lm_48 and lm_53, shikimate and survival data did not match. These samples were excluded from further analysis.

### 3.2 No evidence for known resistance mechanisms in Oregon populations of *L. multiflorum*

The sequence analysis of *EPSPS* in Oregon populations of *L*. *multiflorum* revealed no evidence of mutations at positions 102 or 106, sites shown previously to be associated with the resistance phenotype (NCBI accession numbers MZ418136 and MZ418137). PRHC was included as a positive control for the presence of a functionally relevant amino acid substitution in EPSPS at position 106 that confers resistance. As expected, a mutation was found in *EPSPS* at position 106 from PRHC, causing a proline‐to‐alanine substitution. There were synonymous mutations found among the sequenced individuals from Oregon, but no mutation was found in common among all resistant individuals sequenced and no nonsynonymous mutations were observed.

**FIGURE 3:**
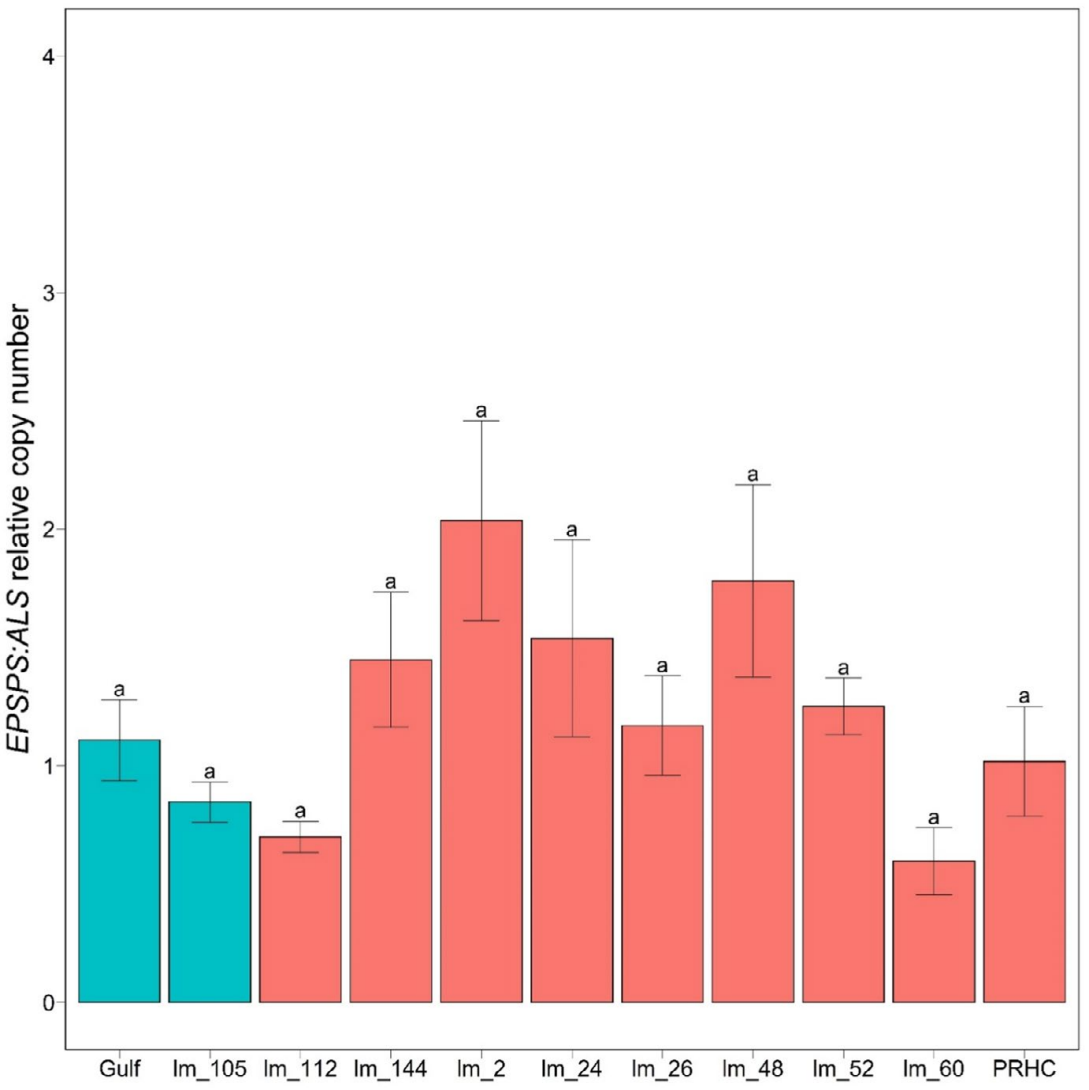
Copy‐number variation of *EPSPS* among *L*. *multiflorum* populations relative to *ALS*. Bars represent standard errors around the mean. Copy‐number variation was quantified using the 2^−ΔΔCt^ method. No significant difference was detected between resistant and susceptible populations. Multiple comparisons were performed using Tukey's contrasts, with the Bonferroni‐corrected p‐values considering an α = 0.05. Green bars indicate susceptible populations, and red bars correspond to resistant populations

**FIGURE 4:**
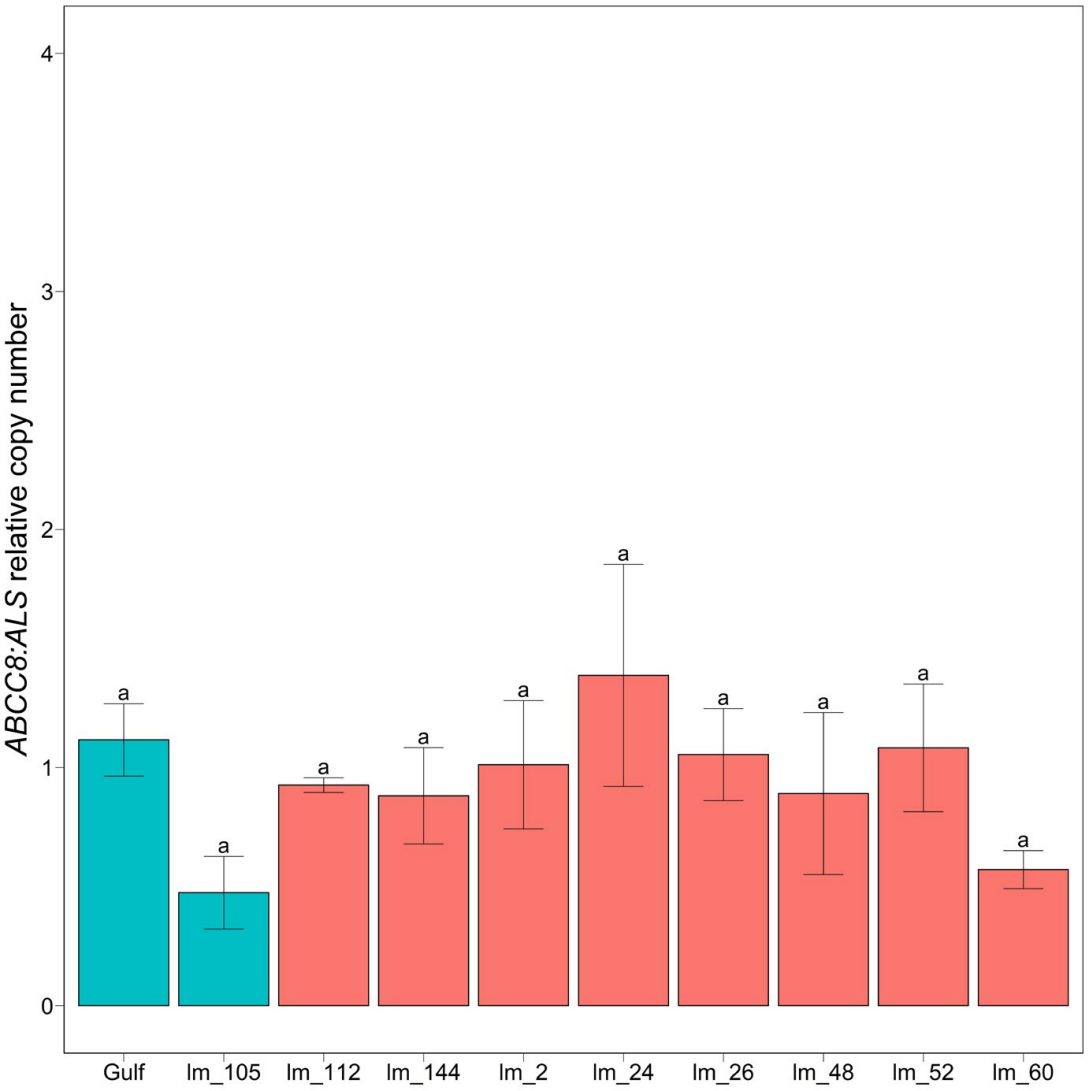
Expression of the ATP‐binding cassette *ABCC8* in two glyphosate‐susceptible populations (green) and eight glyphosate‐resistant (red) populations of *L*. *multiflorum* relative to the housekeeping gene *ALS*. Bars represent standard errors around the mean. Gene expression variation was quantified using the $2^{‐\Delta \Delta C_{t}}$ method. No significant difference was detected between resistant and susceptible populations. Multiple comparisons were performed using Tukey's contrasts, with the Bonferroni‐corrected p‐values considering an α = 0.05

Little variation was observed in the number of *EPSPS* copies across the surveyed populations relative to the housekeeping gene *ALS* (Figure 3). After normalizing to *ALS*, mean and median *EPSPS* copy numbers across Oregon populations relative to Gulf were 1.13 and 0.99, respectively, and no statistically significant differences between resistant and susceptible populations were observed. Moreover, we did not observe differences between resistant and susceptible plants in the expression levels of the ABC transporter *ABCC8* in the Oregon *L*. *multiflorum* populations (Figure 4), with mean and median *ABCC8* expression of 0.93 and 0.81 relative to Gulf. These results reveal that known mechanisms are not responsible for generating glyphosate resistance in *L*. *multiflorum* populations from Oregon, which suggests a novel mechanism controlling resistance.

### 3.3 Little genetic structuring of resistant and susceptible populations

After removing reads with adaptor sequence, without barcodes and restriction enzyme cut sites, and discarding low‐quality reads, the *process_radtags* program retained an average of 407 M (*SE* = 6.7 M) reads per lane that were fed to the *Stacks* pipeline. The average number of reads per sample was approximately 1,800,000 (*SE* = 24,000). The *gstacks* module indicated the mean coverage was 12.5X (min = 9.5; max = 18.1). Following the filtering steps in the *populations* module and vcftools, 2,193 SNPs were retained for population genetic analyses.

**FIGURE 5:**
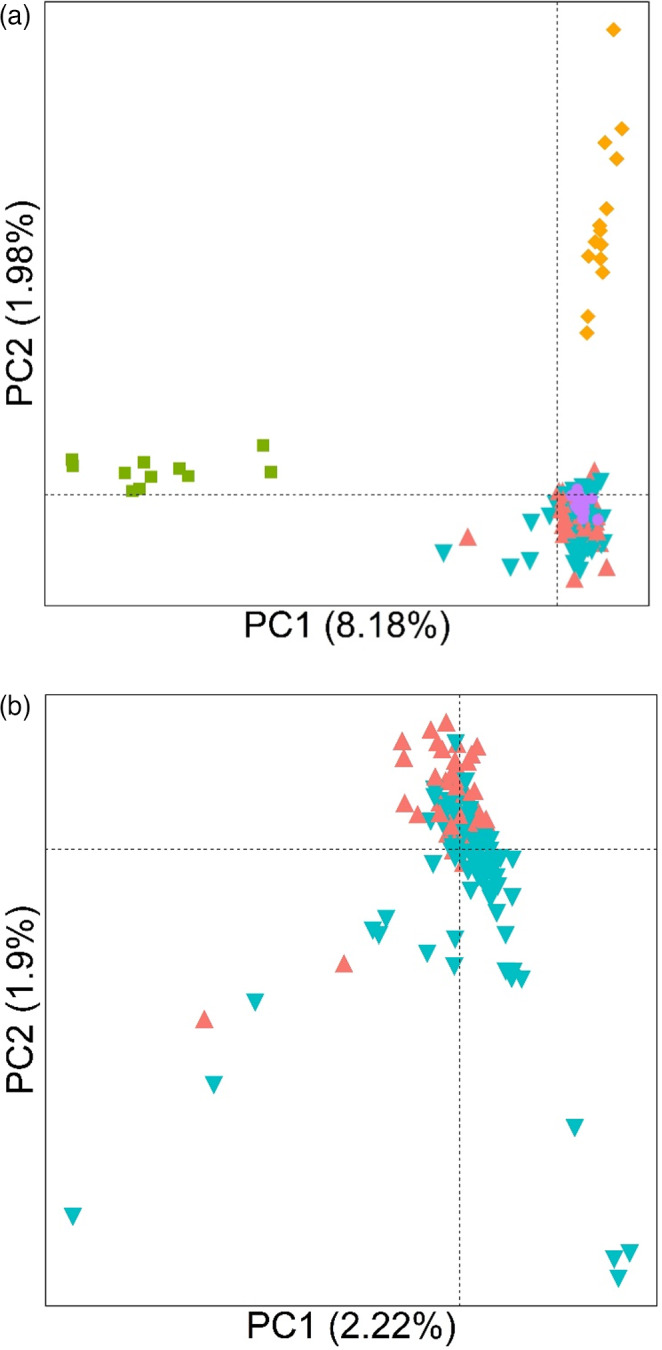
(a) Principal component analysis (PCA) from 2193 SNPs among individuals from Oregon‐resistant (red triangles), Oregon‐susceptible (green, upside down triangles), Gulf (purple, solid circles), perennial (green squares), and PRHC (orange diamonds) *L*. *multiflorum* populations. (b) PCA, including only the Oregon *L*. *multiflorum* populations

Patterns of genetic variation among individuals and populations are structured primarily according to geography and species, rather than their resistance phenotype. In the analysis of the entire dataset, the first principal component mainly reveals differentiation between the two different species: the annual and perennial ryegrass (Figure 5A). Similarly, PC2 largely separates the geographically distant PRHC population from the Oregon populations. Moreover, there is little separation between the resistant and susceptible samples along the first two principal components, suggesting a recent common ancestor of these individuals and/or ongoing gene flow between them. Among the Oregon populations only, PCA explains little of the genetic variation present (the first two principal components explain a combined 4% of the variation) (Figure 5B), and resistant and susceptible samples are not differentiated from each other. Consistent with the PCA, average pairwise F_ST_ indicates little differentiation among populations in Oregon (mean = 0.09, median = 0.09) (Figure S1).

**FIGURE 6:**
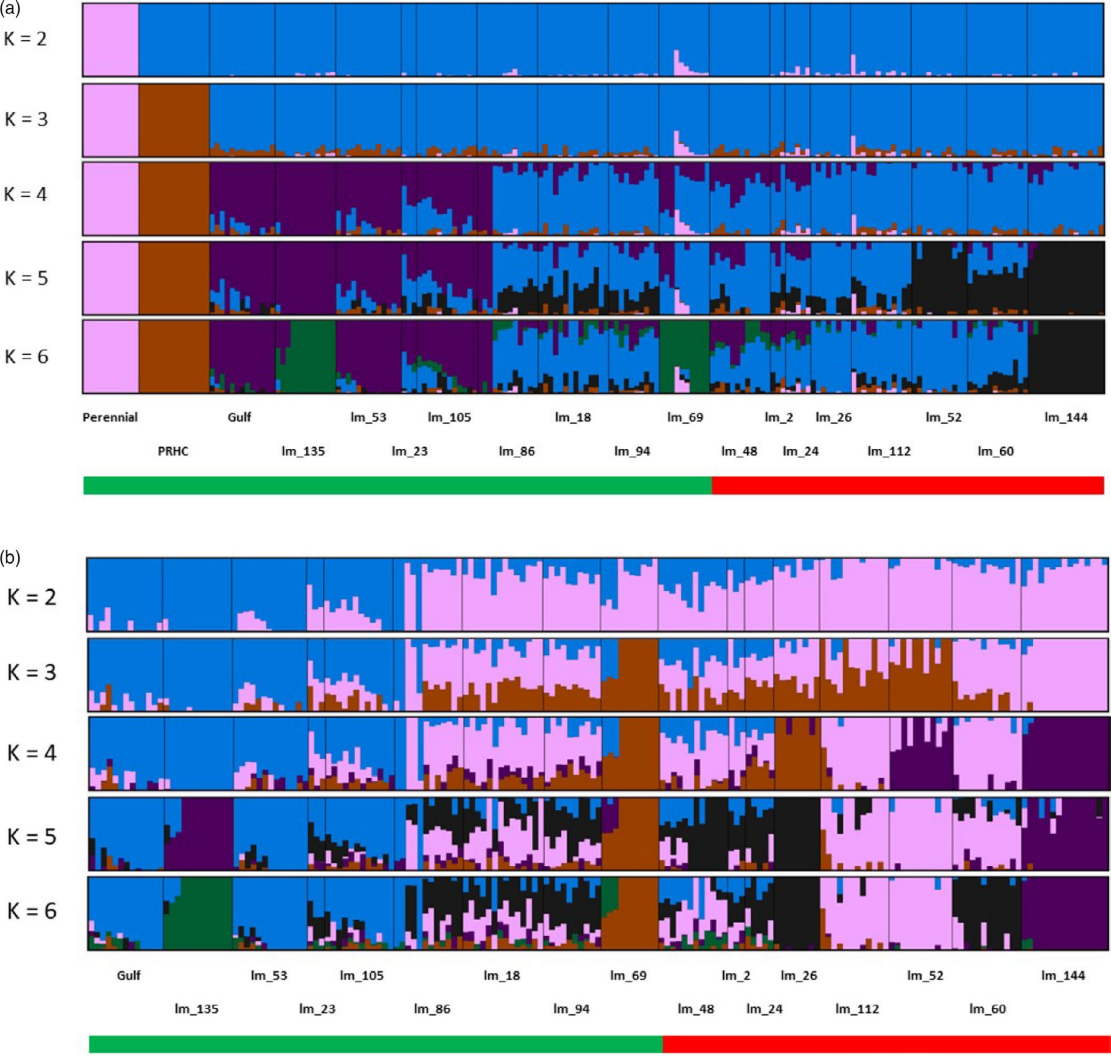
Ancestry coefficients from *Admixture* showing assignment probabilities into *K* = 2 to 6 different clusters. Analysis with the full dataset (a), and only weedy *L*. *multiflorum* (b) populations from Oregon. The green horizontal bar represents glyphosate‐susceptible populations, whereas red represents glyphosate‐resistant

The results from ADMIXTURE are largely consistent with the PCA. The full dataset indicates that most of the *L*. *multiflorum* samples share little ancestry with plants from the perennial species (*L*. *perenne*) (Figure 6A). However, some populations do exhibit shared ancestry with the perennial species (particularly population lm_69). This can potentially be explained by hybridization and introgression between these species, as both species coexist and remain interfertile. Despite their geographical overlap, the shared ancestry between Oregon *L*. *multiflorum* and perennial ryegrass remains limited, perhaps because of differences in flowering time between these species in the field (Chastain et al., 2015; Martinez‐Ghersa et al., 2008).

When *L*. *perenne*, PRHC, and Gulf are removed from the analysis, additional differences are observed among the Oregon populations. Consistent with the PCA, at *K* = *2*, the resistant and susceptible samples are not assigned to separate clusters. Rather, there is extensive shared ancestry among these samples, further supporting a recent common ancestor among all of the Oregon populations. However, at higher values of *K*, multiple distinct groups become evident among the glyphosate‐resistant populations. There is some shared ancestry between the resistant and susceptible populations, as well as unique population structure among some susceptible populations, but there is no evidence that all resistant populations have the same historical pattern of ancestry. These results suggest that glyphosate resistance likely has evolved independently on different genetic backgrounds. Moreover, if resistance evolved once and was shared among populations, we might expect that resistant populations would show reduced levels of genetic diversity. However, we did not observe statistical differences when population genetic estimates (i.e., H*_E_*, π, H_O_, and F_IS_) were compared between the resistant and susceptible populations (Table S3), implying no evidence for founder events that accompanied the rapid evolution of resistance. These analyses provide consistent findings of little overall population structure among Oregon populations of *L*. *multiflorum*, but the presence of modest divergence among resistant populations suggests that resistance evolved on multiple distinct genetic backgrounds.

### 3.4 Patterns of genetic differentiation do not support a single origin of resistance

**FIGURE 7:**
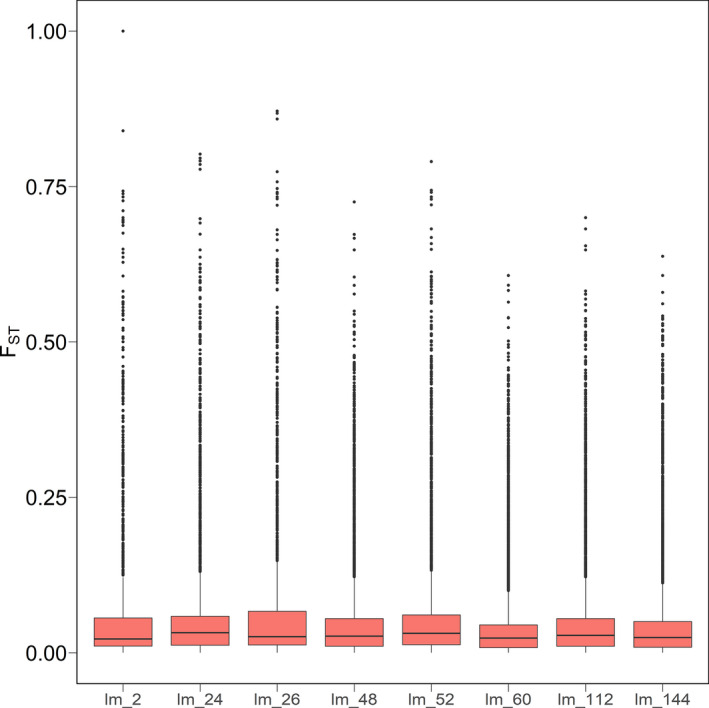
Distribution of F_ST_ values for all SNPs between each glyphosate‐resistant *L*. *multiflorum* population (listed) and each of the susceptible populations. Horizontal lines correspond to the median F_ST_, box heights indicate the lower and upper quartile, and whiskers correspond to 1.5 times the interquartile range. Outliers outside this range are depicted as points

After imputing missing data with LinkImputeR using the thresholds of depth = 4, and missingness = 0.9, the SNP dataset used for inferring patterns of population differentiation (F_ST_) included 28,078 sites, with imputation accuracy of 0.9506. Between each pair of resistant and susceptible populations, overall levels of population differentiation were quite low. Median F_ST_ varied from 0.022 to 0.032, depending on the population, with most of the third quartile of the distribution below 0.067 (Figure 7). Despite the overall low median F_ST_ among the glyphosate‐resistant populations analyzed, on average, 0.86% of the loci revealed F_ST_ above 0.5, with one SNP in locus 23735 reaching F_ST_ of 1 in population lm_2. Overall, there were no loci that were consistently found in the top 1% of the F_ST_ distribution for all resistant and susceptible population pairs. Therefore, we next asked whether there were any shared outliers in comparisons between a single resistant population and each of the susceptible populations. Resistant populations lm_2, lm_24, lm_26, lm_60, lm_112, and lm_144 exhibited 16, 2, 2, 4, 1, and 1 loci, respectively, that were ranked in the top 1% of the distribution for all pairwise comparisons (Table S4). Populations lm_48, lm_52, and lm_60 did not have any SNPs that were consistently ranked in the top 1% of the F_ST_ distribution that were shared among all susceptible populations. Only population lm_2 exhibited F_ST_ of 1 (Figure 7). The predicted proteins encoded by genes found on the contigs containing these loci included transmembrane transporters, protein kinase (L‐type lectin‐domain containing receptor kinase IX.2‐like), anion transporters (GABA transporter 1), and enzymes previously reported to be able to metabolize herbicides (P450s), among other hypothetical proteins of unknown function (Table S5).

## 4 DISCUSSION

The evolution of herbicide resistance is a growing challenge to broad acreage agricultural systems that depend on herbicides for weed management. In many cases, alternate control methods are costly or unavailable, and chemical companies have not introduced many new herbicides over the past few decades. Herbicide resistance in weeds is a clear example of rapid adaptation caused by repeated, strong selection pressures induced by human intervention. Our findings reveal multiple instances of the evolution of glyphosate resistance in *L*. *multiflorum* across different fields in Oregon. Despite frequent examples of resistance evolving due to mutations that impact the target site, our sequence and population genomic analyses imply a more complex history of resistance.

Amino acid substitutions at positions 102 and 106 in EPSPS have been shown to confer resistance to glyphosate in many different weed species and populations. For example, target‐site mutations in *EPSPS* have been detected in *L*. *multiflorum* (Brunharo & Hanson, 2018) and *E*. *colona* (Morran et al., 2018) in California, *Chloris virgata* (Sw.) in Australia (Ngo et al., 2017), *Amaranthus tuberculatus* (Moq.) J. D. Sauer in Mississippi (Nandula et al., 2013), and others. Although Karn and Jasieniuk (2017) also found that all resistant populations of *L*. *multiflorum* sampled in California exhibited at least one allele with missense mutations at position 106, in some cases, resistant individuals within those populations did not carry a function‐altering mutation in *EPSPS*. Thus, although mutations in *EPSPS* were commonly associated with the evolution of resistance, multiple glyphosate resistance mechanisms appear to have evolved in those populations. By contrast, in the resistant Oregon *L*. *multiflorum* populations we sequenced, no missense mutations in *EPSPS* were found. Therefore, despite the convergence on this single target‐site mutation across many different weed species from geographically widespread areas, in Oregon *L*. *multiflorum*, additional mechanisms must be involved in conferring resistance.

One additional known glyphosate resistance mechanism is increased copy number of the *EPSPS* gene. Copy‐number variation can lead to increased dosage of EPSPS protein in plant cells, requiring higher concentrations of glyphosate to inhibit the enzyme (Powles, 2010). Copy‐number differences in *EPSPS* that confer glyphosate resistance have been detected in several weed species. For example, in *Amaranthus palmeri* S. Watson, resistant plants had up to 160‐fold more copies of *EPSPS* than susceptible individuals, likely due to extrachromosomal circular DNA (Koo et al., 2018). In addition, resistant *Bassia scoparia* (L.) A. J. Scott plants had up to 10 copies of the gene (Gaines et al., 2016), likely mediated by a mobile genetic element (Patterson et al., 2019). Lastly, *EPSPS* duplication associated with a missense mutation in the *EPSPS* gene has been observed in the allotetraploid grass species *Poa annua* L. (Brunharo et al., 2018). These previous studies on glyphosate resistance suggest that weeds have evolved different pathways to cope with the herbicide and that parallel glyphosate resistance evolution is not uncommon. However, our results do not support a role for copy‐number variation in *EPSPS* impacting glyphosate resistance in Oregon populations of *L*. *multiflorum* (Figure 3), again suggesting that an unknown mechanism is responsible for the resistance phenotype.

In addition to *EPSPS*, a plasma membrane‐localized ABC transporter recently has been identified to confer glyphosate resistance in an *E*. *colona* population from Australia (Pan et al., 2021). Data suggest that this transporter enhances glyphosate efflux from the cytoplasm into the apoplast. Further functional characterization was confirmed by transforming several plants to overexpress the *ABCC8* gene, which conferred tolerance to field rates of the herbicide. Our results show no evidence of differences in *ABCC8* expression between resistant and susceptible populations of *L*. *multiflorum* in Oregon. Thus, despite the broad spectrum of genetic changes capable of conferring glyphosate target‐site resistance, an uncharacterized mechanism, likely involving a non‐target‐site basis, appears to confer resistance in Oregon populations of *L*. *multiflorum*.

In addition to determining the mechanism for resistance, an additional goal of this study was to investigate the evolutionary origins and processes that led to the spread of glyphosate resistance among fields throughout Oregon. There are at least three different processes that could explain the presence of multiple glyphosate‐resistant *L*. *multiflorum* populations found in different fields across Oregon. First, there could be a single origin of resistance, followed by human‐mediated seed dispersal of the resistance alleles across the range. Alternatively, there could be a single origin, followed by natural pollen flow among neighboring populations. Finally, there could be multiple origins of resistance due to independent mutations in the same or different genes. Our results favor the third explanation. If there was a single origin of resistance, then we would expect all resistant individuals to group together in our population genetic analyses. However, the PCA showed no clustering of resistant and susceptible individuals. Similarly, at *K *= 2, there was no evidence of distinct ancestry patterns associated with all resistance individuals. In addition, we likely can rule out a single origin followed by gene flow among populations, because *L*. *multiflorum* was introduced to Oregon in the early 1900s, and glyphosate did not become adopted as a widespread herbicide until the year 2000 (Benbrook, 2016). Therefore, it is unlikely that there would have been sufficient time for glyphosate resistance gene(s) to spread naturally via gene flow across this expansive region, but simulations would be needed to determine the spatial scale over which a resistance allele could spread during this time. By contrast, at higher values of *K*, distinct patterns of ancestry emerged in many of the resistant populations. Given that these populations are all resistant to the same herbicide but have different patterns of ancestry provides strong evidence that resistance originated or was selected from the standing variation in multiple populations. Confirming this hypothesis will require future characterization of the gene or genes that confer resistance.

Consistent with a multiple‐origins model, our analysis of genetic divergence across the genome of *L*. *multiflorum* fails to find loci that were repeatedly differentiated between resistant and susceptible populations. Under a model of a single origin of resistance and repeated, strong, and recent selection, we would expect to find variants at high frequency in the resistant individuals that are at low frequency in the susceptible plants. This should be manifest as high F_ST_ at SNPs linked to the mutation conferring resistance. Moreover, this locus should show consistently high F_ST_ between each pair of resistant and susceptible populations. However, we found no SNPs in our dataset that were repeatedly at high F_ST_ between different pairs of resistant and susceptible populations. Although the obligate outcrossing, annual life history of *L*. *multiflorum*, and the reduced‐representation genotyping approach used here limit the genomic resolution and the extent of linkage disequilibrium (LD) in the dataset, strong and recent natural selection should result in long blocks of LD between SNPs reasonably tightly linked to functional mutations. By contrast, it is likely that the diverse patterns seen in these resistant Oregon populations are due to mutations (maybe at different loci) that pre‐dated the widespread use of glyphosate and segregated at low frequency in the ancestral population. To test this scenario, a different approach aimed at detecting selection on standing genetic variation would be better suited than one that searches for shared outliers. However, the lack of shared outliers is consistent with our analyses of population structure, further supporting the conclusion that resistance does not have a single origin in these Oregon populations. The assembly of a reference genome for *L*. *multiflorum* may aid in future studies as well. Given the apparent complexity of the origin of resistance in these populations, future studies that sequence whole genomes from these populations will be necessary to test these alternate hypotheses.

Even though no outliers were consistently found between all pairs of resistant and susceptible individuals, we did find SNPs with high F_ST_ that were in common between individual resistant populations and each of the susceptible populations. Gene annotation and ontology analyses of genomic contigs containing these genes identified several molecular functions potentially associated with resistance. For example, we detected genes involved in the detoxification of xenobiotics, most notably cytochrome P450 genes. Genes in this family have been suggested to be involved in glyphosate resistance by enhancing herbicide degradation in other populations of *L*. *multiflorum*, *L*. *rigidum* Gaudin, and *L*. *perenne* outside of Oregon (Suzukawa et al., 2021). In addition, we identified genes involved in transmembrane transport (Table S5). Vacuolar sequestration of glyphosate has been suggested to confer resistance in other species (Peng et al., 2010). Although much research has been conducted to elucidate non‐target‐site resistance mechanisms to glyphosate in *L*. *multiflorum* and other weed species, the genetic and molecular mechanisms remain largely unknown (Suzukawa et al., 2021).

This research suggests that glyphosate resistance evolved multiple times in *L*. *multiflorum* populations from Oregon. Elucidating the molecular mechanisms of herbicide resistance is crucial for the improvement in weed management practices. Although target‐site resistance has been described frequently for many herbicides with different mechanisms of action, the underlying molecular characteristics that confer non‐target‐site resistance remain largely unknown (Baucom, 2016; Suzukawa et al., 2021). Although the resistance phenotype may have multiple origins, our data suggest that resistance is likely to have a simple genetic basis in all cases. We consistently found qualitative, rather than quantitative, differences in shikimate accumulation between plants from resistant and susceptible populations that were highly correlated with survival following glyphosate treatment. Thus, identification of the genetic changes involved in resistance evolution will allow the development of quick herbicide resistance diagnostics in the laboratory and in the field. The rapid diagnosis of herbicide resistance will allow the development of measures that slow down, or preferably prevent, the introduction of resistance alleles to new areas, reducing the long‐term costs associated with herbicide resistance. Finally, because non‐target‐site resistance may confer resistance to herbicides from different chemical groups (i.e., generalist resistance mechanisms; Comont et al., 2020), a more in‐depth understanding of these mechanisms will allow better utilization of herbicides to manage the spread of resistance.

## CONFLICT OF INTEREST

The authors have no conflict of interest to declare.

## AUTHOR CONTRIBUTIONS

CAB designed the research and performed the experiments. CAB and MAS performed the statistical analysis, data interpretation, and manuscript preparation. CAB and MAS contributed equally.

## Supporting information

Supplementary Material

## Data Availability

The genotype‐by‐sequencing data used in this study are available at NCBI SRA PRJNA739185. EPSPS and ABCC8 sequences are available at NCBI (accession numbers MZ418136 and MZ418137). Raw data used for the EPSPS amplification and ABCC8 expression are available at https://github.com/caiobrunharo/popgen_ryegrass.
